# Knowledge, Attitudes, and Practices of Thai Slaughterhouse Personnel Regarding Bovine Tuberculosis Surveillance: A Multi-Regional One Health Assessment

**DOI:** 10.3390/vetsci12020135

**Published:** 2025-02-06

**Authors:** Pongpon Homkong, Sukolrat Boonyayatra, Napat Harnpornchai, Terdsak Yano, Warangkhana Chaisowwong

**Affiliations:** 1 Faculty of Veterinary Medicine, Chiang Mai University, Chiang Mai 50100, Thailand; pongpon_h@cmu.ac.th; 2 Veterinary Service, Department of Public Health and Environmental, Chiang Mai Municipality, Chiang Mai 50300, Thailand; 3 College of Veterinary Medicine, Long Island University, Brookville, NY 11548, USA; sukolrat.boonyayatra@liu.edu; 4 Faculty of Economics, Chiang Mai University, Chiang Mai 50200, Thailand; napat.h@cmu.ac.th

**Keywords:** slaughterhouse personnel, disease surveillance, questionnaire, cross-sectional study, Thailand, zoonotic disease

## Abstract

This cross-sectional study investigated knowledge, attitudes, and practices (KAP) regarding bovine tuberculosis surveillance and analyzed factors influencing KAP among Thai slaughterhouse personnel, utilizing a One Health approach. A validated questionnaire was administered to 208 participants across five geographical regions of Thailand. Statistical analysis revealed moderate mean scores for knowledge (5.28/10), attitudes (38.55/65), and practices (34.62/50). Significant differences were observed in knowledge scores across education levels (F = 3.427, *p* = 0.005) and job positions (F = 4.562, *p* = 0.011), with higher education and managerial positions being associated with better performance. Path analysis demonstrated positive correlations among KAP components (*p* < 0.05), with the strongest correlation between attitudes and practices (r = 0.543). The most significant risk factors for poor practices were identified as lack of training (OR = 2.76, 95% CI: 1.45–5.24, *p* = 0.002) and inadequate tuberculosis screening (OR = 2.31, 95% CI: 1.18–4.52, *p* = 0.015). The Knowledge–Practice gap index of −31.14 indicated substantial discrepancy between knowledge and implementation. Developing targeted training programs is needed by focusing on knowledge enhancement and awareness-building, as well as fostering inter-agency collaboration in line with the One Health approach enhance the efficiency of bovine tuberculosis surveillance in Thailand.

## Simple Summary

Bovine tuberculosis (bTB) surveillance in slaughterhouses plays a crucial role in protecting both animal and public health. This study investigated the knowledge, attitudes, and practices (KAP) of personnel working in Thai slaughterhouses regarding bTB surveillance. We surveyed 208 workers across Thailand’s five geographical regions, including facility owners, managers, meat inspectors, and operational staff. Our findings reveal moderate levels of knowledge and varying attitudes toward surveillance practices. Importantly, we found that lack of training and insufficient health screening were major risk factors affecting surveillance effectiveness. Workers with higher education levels and those in managerial positions showed better understanding of surveillance requirements. This study highlights the need for comprehensive training programs and stronger collaboration between different sectors involved in disease control. These findings can help improve bTB surveillance systems in Thailand and provide insights for other countries facing similar challenges in controlling zoonotic diseases.

## 1. Introduction

Bovine tuberculosis (bTB), caused by *Mycobacterium bovis*, represents a significant zoonotic threat at the human–animal interface, particularly in emerging economies like Thailand. This bacterial pathogen, a member of the *Mycobacterium tuberculosis* complex [1], creates a substantial burden through its dual impact on animal productivity and public health. In livestock, bTB causes chronic infections leading to reduced productivity, increased mortality, and significant economic losses [2]. The World Health Organization (WHO) estimates that *M. bovis* was responsible for approximately 142,000 new human tuberculosis cases in 2017, resulting in over 12,500 deaths, predominantly in Africa and South Asia [3]. Traditional farming practices [4] and complex food supply chains in Thailand [5] create unique challenges for bTB surveillance. The integration of slaughterhouse surveillance within the national disease control strategy represents a critical point for the early detection and prevention of bTB transmission [6].

Clinical manifestations of bTB in cattle include chronic cough, weight loss, and reduced milk production, although many infected animals may remain asymptomatic [6]. The disease primarily spreads through aerosol transmission in enclosed spaces, making slaughterhouse workers particularly vulnerable to infection [1]. In Thailand, where traditional farming practices persist [4] and livestock movement patterns are complex [5], recent epidemiological studies revealed concerning bTB prevalence rates of 14–22% in dairy cattle [7], with similar findings observed in slaughterhouses across the region. The combination of high humidity, limited ventilation, and prolonged close contact between workers and potentially infected animals creates an environment conducive to disease spread [2,6]. These conditions highlight the critical need for effective surveillance and control measures in slaughterhouse settings [6].

Thailand’s Department of Livestock Development has implemented a mandatory surveillance program requiring biannual testing and elimination of infected dairy cattle aged one year or older using the caudal fold Single Intradermal Test (SIT) [8]. While this represents a systematic approach to disease control, the SIT has recognized limitations, particularly in detecting early- or late-stage infections [9]. Current research suggests that integrating multiple diagnostic methods, including the gamma interferon release assay (IGRA), significantly improves disease detection capabilities [9,10]. Unlike the SIT, IGRA demonstrates improved sensitivity and specificity for detecting latent infections, making it a widely accepted diagnostic tool in veterinary practices [9]. Additionally, the simple and comparative cervical intradermal tests are recognized as more sensitive and specific intradermal methods compared to the caudal fold test, further enhancing diagnostic accuracy in field settings [9]. Recent epidemiological studies in northern Thailand revealed concerning bTB prevalence rates of 14–22% in dairy cattle [7], with similar findings observed in slaughterhouses across the region. This highlights the need for robust surveillance efforts targeting these facilities to mitigate zoonotic transmission risks.

Slaughterhouse-based surveillance has emerged as a cornerstone of bTB control strategies globally, particularly in countries that are yet to achieve complete disease eradication [11]. This approach offers several distinct advantages over farm-level testing, notably the ability to detect pathological lesions that might otherwise go unnoticed [12]. Post-mortem inspection provides superior diagnostic validity for tuberculosis [13], enabling more accurate disease detection and surveillance. The systematic nature of slaughterhouse monitoring facilitates comprehensive disease surveillance through multiple mechanisms: enhanced detection efficiency [14], population-level prevalence monitoring [15], and early warning capabilities for disease outbreaks. Beyond lesion identification, slaughterhouses serve as crucial data collection points, providing valuable specimens for laboratory analysis, generating epidemiological data for relevant authorities, and enabling trace-back investigations to identify disease origins [16]. However, slaughterhouse surveillance is not without limitations, such as difficulty detecting non-visible lesions, reliance on trained personnel, and delays in diagnosis due to operational gaps. Addressing these challenges is essential to maximize its effectiveness in bTB control.

Slaughterhouse surveillance data provides valuable insights for evaluating farm-level control measures and informing evidence-based policy decisions for disease control and eradication strategies [11,13,17]. The quality of this surveillance system, however, depends heavily on various operational factors, including time allocation, workload management, and inspector competency [16,18]. Recent studies in Southeast Asian contexts have highlighted how cultural practices and workforce demographics influence surveillance effectiveness [19]. This understanding has led to increased recognition that knowledge, attitudes, and practices (KAP) of slaughterhouse personnel are fundamental determinants of surveillance system efficiency [14]. The integration of these behavioral aspects with technical surveillance requirements becomes particularly crucial in Thailand’s context, where traditional practices often intersect with modern food safety standards [8].

The Knowledge, Attitudes, and Practices (KAP) framework has emerged as an essential tool for evaluating and strengthening disease surveillance systems [20,21,22]. This approach is particularly valuable within the One Health paradigm, where understanding the interconnections between human, animal, and environmental health is crucial for effective disease control [17]. In occupational settings such as slaughterhouses, KAP studies have demonstrated how worker behaviors influence zoonotic disease transmission risks [23]. Comprehensive reviews have shown that KAP components significantly impact both individual compliance with safety protocols and overall surveillance system effectiveness [24]. Furthermore, studies have identified correlations between KAP levels and successful implementation of disease control measures including in Thailand [20].

Multiple cross-sectional studies from various nations have revealed concerning patterns in slaughterhouse workers’ knowledge and practices. Studies from Nigeria [25] and Ethiopia [26] highlighted significant gaps in workers’ understanding of bTB transmission. Research conducted in Bangladesh [27], Nepal [28], and Tanzania [29] has consistently demonstrated disparities between knowledge levels and actual practices. These studies have identified that KAP measures strongly correlate with educational background, years of professional experience, and training received. The evidence suggests that providing comprehensive training and information about disease reporting systems in abattoirs is crucial for reducing the gap between individual knowledge and system capabilities, particularly in enhancing tuberculosis reporting systems.

The identification of risk factors influencing tuberculosis transmission and KAP patterns is crucial for developing effective disease control strategies. Epidemiological risk assessments by Humblet et al. [30] identified key variables including herd size, animal purchases, and wildlife exposure as significant predictors of disease transmission. These findings align with comprehensive studies from the United Kingdom and Ireland [31]. Social and cultural factors significantly impact farmers’ KAP in managing bTB, as demonstrated by research in England [32]. Environmental and agricultural management practices have also been shown to influence disease occurrence patterns in various settings [33].

The relationship between KAP and bTB transmission risk factors in slaughterhouse settings requires further investigation. Research suggests that integrating slaughterhouse surveillance data with farm-level epidemiology can enhance our understanding of disease dynamics [34]. Recent studies emphasize the importance of developing comprehensive monitoring systems that incorporate social and behavioral elements [35]. While previous research has examined bTB epidemiology in Thailand, including prevalence assessment in the northern region [10], there remains limited evidence linking KAP to disease surveillance effectiveness in slaughterhouses. The Thai Animal Disease Early Warning System study [4] emphasized human resource development and disease reporting, aligning with integrated cross-sector collaboration goals [36].

This study aims to contribute significantly to the development of protocols for enhanced bTB surveillance, ensuring their practical applicability in slaughterhouse contexts. The findings will enhance understanding of bTB surveillance in Thai slaughterhouses, with implications for developing targeted training programs and disease prevention strategies [37]. Understanding the correlation between preventable disease factors and KAP can improve existing surveillance methods [38]. These results will inform policy development and early intervention measures for bTB control in Thailand [39], with potential applications for surveillance programs across Southeast Asia [40].

This study addresses the knowledge gap regarding KAP in bTB surveillance at Thai slaughterhouses through four specific objectives:

1. Evaluate the competencies, beliefs, and practices regarding bTB surveillance among personnel in Thai slaughterhouses.
2. Examine factors influencing KAP in slaughterhouse bTB surveillance.
3. Analyze the relationship between KAP and bTB transmission risk factors.
4. Develop protocols for implementing bTB surveillance systems in Thai slaughterhouses using the One Health approach.

## 2. Materials and Methods

### 2.1. Study Design and Area, Data Collection, and Data Sampling 

A cross-sectional study was conducted across Thailand’s slaughterhouse network from January to December 2023, encompassing all five major geographical regions of Thailand (Northern, Central, Northeastern, Eastern, and Southern) to ensure comprehensive national representation. The sampling frame comprised 462 registered cattle and buffalo slaughterhouses, as documented by the Department of Livestock Development in 2022. These facilities represented diverse operational scales, from small local establishments to large industrial complexes, providing a representative overview of Thailand’s meat processing infrastructure.

This study targeted three distinct categories of slaughterhouse personnel: facility owners and managers, veterinary meat inspectors, and operational staff involved in ante- and post-mortem animal handling. All participants were required to be aged 18 years or above, Thai-speaking, actively employed at the selected facilities, and willing to participate in the study. Sample size was determined using the formula n = Z^2^_1−a/2_ P(1 − P)/d^2^, where Z_1−a/2_ represented 1.96 at 95% confidence level, P was set at 50% for maximum sample size (expected proportion with adequate KAP), and d represented 7% absolute precision. The calculated minimum sample size of 196 was increased to 208 to account for potential non-response.

To ensure representative sampling, a multi-stage random sampling approach was implemented. Initially, the country was stratified into five geographical regions, followed by random selection of provinces within each region using probability proportional to size. Slaughterhouses within selected provinces were then chosen through systematic random sampling. Within each selected facility, purposive sampling of personnel was conducted to ensure adequate representation across all staff categories, minimizing selection bias while maintaining the representation of different facility types and personnel roles.

A structured questionnaire was developed based on World Organization for Animal Health (WOAH) standards for bovine TB monitoring and previous KAP studies in veterinary public health. The questionnaire consisted of four primary sections: participant sociodemographic characteristics, knowledge assessment through ten multiple-choice questions about bovine TB surveillance, attitude evaluation using a 13-item 5-point Likert scale, and practice assessment via ten questions on a 5-point Likert scale. The knowledge section covered disease etiology, transmission, clinical manifestations, and prevention methods. Attitude items evaluated opinions on surveillance importance, willingness to report suspected cases, and control measure efficacy. The practice section assessed compliance with standard operating procedures, personal protective equipment usage, and ante- and post-mortem examination protocols.

To ensure linguistic and cultural appropriateness, the questionnaire underwent rigorous translation and validation processes. Professional translators conducted forward and backward translation between English and Thai, followed by pre-testing on twenty veterinary students and slaughterhouse employees not included in the final sample. Content validation was performed by five experts in epidemiology, veterinary public health, and bovine tuberculosis, with items scoring above 0.80 on the Content Validity Index being retained. Internal consistency was confirmed through Cronbach’s alpha analysis, yielding strong reliability coefficients for knowledge (0.85), attitude (0.82), and practice (0.88) sections. Data collection was conducted through in-person and online interviews by experienced veterinary researchers, with observational checklists used where possible to verify self-reported behaviors.

### 2.2. Data Analysis

Data management and statistical analyses were conducted using a combination of specialized software packages: Microsoft Excel 2019 for initial data organization, R version 4.1.0 for comprehensive statistical testing, and Tableau 2021.2 for data visualization. The analytical framework followed a systematic multi-stage approach designed to address the study’s objectives comprehensively. Descriptive statistics, including frequencies, percentages, means, and standard deviations, were calculated for socio-demographic variables and KAP scores. For knowledge assessment, the frequency and percentage of correct responses were computed for each item, and total scores were calculated to evaluate respondents’ overall knowledge levels. Attitude and practice levels were assessed through mean scores, standard deviations, and frequency distributions of both individual items and composite scores.

Statistical comparisons between groups employed both parametric and non-parametric methods based on data distribution characteristics. Independent t-tests were conducted to compare KAP scores between binary categories such as gender [41], while one-way ANOVA was utilized for comparing scores across multiple groups [42], such as education levels and job roles. Post hoc analyses using Tukey’s HSD test were performed following significant ANOVA results to identify specific group differences [43]. For categorical variables, chi-square tests were employed to examine associations [44], with Fisher’s exact test applied when cell frequencies did not meet chi-square test assumptions [45].

Correlation analysis utilized Pearson’s correlation coefficient to examine relationships between continuous variables, particularly focusing on associations between knowledge, attitude, and practice scores [46]. Multiple linear regression analysis was performed to identify factors influencing KAP scores, incorporating independent variables such as age, education level, work experience, and training received [47]. The regression models were evaluated for assumptions including normality, homoscedasticity, and multicollinearity [48]. To examine variables influencing correct practices in bovine tuberculosis surveillance, a logistic regression analysis was conducted, presenting results as Odds Ratios (OR) with 95% Confidence Intervals [49].

The knowledge gap analysis was implemented to identify discrepancies across knowledge, attitudes, and practices domains [50]. The Knowledge–Practice (K–P) gap index was computed to quantify the disparity between knowledge and implementation [51]. To analyze the complex interrelationships among KAP variables, Structural Equation Modeling (SEM) was applied using AMOS version 26 [52]. The SEM model incorporated both measurement and structural components, with model fit assessed using standard indices including Comparative Fit Index (CFI), Tucker–Lewis Index (TLI), Root Mean Square Error of Approximation (RMSEA), and Standardized Root Mean Square Residual (SRMR).

Qualitative data from open-ended responses underwent thematic analysis using a systematic coding approach. Initial codes were generated inductively from the data, followed by theme development and refinement through iterative review processes. To ensure analytical rigor, coding was conducted independently by two researchers, with discrepancies resolved through discussion. The integration of quantitative and qualitative findings provided a comprehensive understanding of KAP patterns and their determinants in the context of bovine tuberculosis surveillance. All statistical tests were conducted at a 5% significance level, with appropriate adjustments for multiple comparisons using Bonferroni correction where necessary. Missing data were handled through multiple imputation techniques when appropriate, ensuring robust analysis while maintaining data integrity.

This research was conducted in accordance with international ethical guidelines, with approval from the Human Research Ethics Committee of Chiang Mai University (Project Number: HS3/2566). All participants provided written or electronic informed consent after receiving comprehensive information about the study. Data protection measures included removing personal identifiers, secure storage of research materials, and restricted data access. The questionnaire underwent cultural and linguistic adaptation following WHO guidelines, with local stakeholder consultation and bilingual research team members ensuring appropriate translation. Data collection in slaughterhouse settings was scheduled to minimize workplace disruption, with interviews conducted in private spaces. The study maintained ongoing communication with facility management and included plans for sharing findings with participating facilities.

## 3. Results

This study comprised 208 participants from slaughterhouse facilities across Thailand’s five geographical regions, with a response rate of 94% (208/221) from eligible participants. Demographic analysis revealed a predominantly male workforce (64.90%, *n* = 135), with females constituting 34.13% (*n* = 71), and 0.96% (*n* = 2) preferring not to specify their gender. The mean age of participants was 45.37 years (SD = 10.52), reflecting a workforce predominantly in their middle-career phase. Educational attainment analysis demonstrated a well-educated workforce, with the majority holding bachelor’s degrees (65.87%, *n* = 137). The complete demographic characteristics of the study population are presented in Table 1.

The knowledge assessment revealed a moderate overall understanding of bovine tuberculosis and surveillance practices. The attitude assessment showed moderately positive attitudes toward bTB surveillance, while practice evaluation demonstrated moderate compliance with recommended surveillance procedures. The overall scores for knowledge, attitude, and practice assessments are presented in Table 2.

The knowledge assessment revealed a moderate overall understanding of bovine tuberculosis and surveillance practices, with a mean score of 5.28 out of 10 (SD = 2.72). Analysis of individual knowledge items showed varying levels of understanding across different aspects of bTB surveillance. As shown in Table 3, the highest correct response rate is observed for the question “Is bovine tuberculosis a severe disease in humans?” (60.10%, n = 125), while the lowest correct response rate is for “Who can first detect cattle with bovine tuberculosis?” (46.63%, n = 97).

The attitude assessment yielded a mean score of 38.55 out of 65 (SD = 10.21), indicating moderately positive attitudes toward bTB surveillance. A detailed analysis of the attitude scores, presented in Table 4, shows that the highest mean score (3.21) was observed for the statement “Disease surveillance in slaughterhouses can help prevent infection transmission to humans”, while the lowest scores (2.76) were recorded for statements regarding surveillance system implementation and government financial support.

Practice evaluation revealed a mean score of 34.62 out of 50 (SD = 7.58), with 57.69% of participants demonstrating moderate compliance with recommended surveillance procedures. As illustrated in Table 5, the highest compliance was observed in “Accepting animals with complete certificates and history” (mean = 3.69), while lower scores were noted for preventive health behaviors such as “You still go to work even when you are sick” (reverse scored, mean = 2.85).

Further analysis of knowledge scores demonstrated significant differences across education levels (F = 3.427, *p* = 0.005) and job positions (F = 4.562, *p* = 0.011). Specifically, owners and managers showed significantly higher knowledge scores compared to general staff (*p* = 0.008), as detailed in Table 6.

A correlation analysis, summarized in Table 7, revealed significant positive associations among knowledge, attitude, and practices (*p* < 0.05), with the strongest correlation between attitudes and practices (*r* = 0.543). A multiple linear regression analysis demonstrated that attitude scores (*β* = 0.478, *p* < 0.001), knowledge levels (*β* = 0.186, *p* < 0.001), and years of work experience (*β* = 0.134, *p* < 0.001) were significant predictors of practice scores, collectively explaining 34.2% of the variance in practice behaviors (*R^2^* = 0.342, *F*(3204) = 35.321, *p* < 0.001).

Logistic regression analysis revealed that knowledge score (OR: 1.18, 95% CI: 1.02–1.37, *p* = 0.029) and attitude score (OR: 1.06, 95% CI: 1.02–1.11, *p* = 0.005) were significantly associated with good practice (Table 8). Other factors including age, education level, work experience, income, and training received showed no significant association with good practice (*p* > 0.05).

The Structural Equation Modeling (SEM) results, detailed in Table 9, demonstrated good fit indices (CFI = 0.891, TLI = 0.885, RMSEA = 0.068, SRMR = 0.059). Path analysis revealed significant direct effects of knowledge on attitudes (β = 0.523, *p* < 0.001) and practices (β = 0.318, *p* < 0.001), as well as attitudes on practices (β = 0.456, *p* < 0.001). Factor loadings for latent variables ranged from 0.52 to 0.78 for knowledge, from 0.61 to 0.83 for attitudes, and from 0.57 to 0.76 for practices, indicating strong construct validity.

The Knowledge–Practice gap analysis revealed a K–P gap index of −31.14, indicating a substantial discrepancy between knowledge levels and actual implementation of surveillance practices. A logistic regression analysis of risk factors, presented in Table 10, revealed that inadequate training was the most significant risk factor (OR = 2.76, 95% CI: 1.45–5.24, *p* = 0.002), followed by the absence of tuberculosis screening (OR = 2.31, 95% CI: 1.18–4.52, *p* = 0.015) and insufficient health examinations (OR = 1.87, 95% CI: 1.02–3.42, *p* = 0.043). Demographic factors, including age (OR = 1.02, 95% CI: 0.99–1.05, *p* = 0.142) and gender (OR = 1.45, 95% CI: 0.78–2.69, *p* = 0.238), showed no significant association with transmission risk.

## 4. Discussion

The primary objective of this research was to assess the Knowledge, Attitudes, and Practices (KAP) regarding bovine tuberculosis surveillance among Thai slaughterhouse workers and investigate the associated risk factors. Theses findings reveal significant patterns in workforce capabilities and system deficiencies that substantially impact Thailand’s disease monitoring efforts. The moderate mean knowledge score of 5.28 out of 10, as shown in Table 2, indicates considerable room for improvement in workers’ understanding of bovine TB surveillance. This finding aligns with research by Cadmus et al. [22] in Nigeria, where slaughterhouse personnel demonstrated limited knowledge of TB transmission in cattle. However, these results diverge from findings in Ethiopia by Fekadu et al. [23], where workers showed high disease awareness, but limited understanding of zoonotic risks. Additionally, research by Mathewos et al. [53] reported higher knowledge levels (73.3%) regarding bTB transmission from bovines to humans among Ethiopian slaughterhouse workers. These disparities likely reflect differences in national training programs and educational initiatives [16,41]. The significant association between education levels and knowledge scores (*p* = 0.005) in this study, as presented in Table 5, emphasizes the crucial role of formal education in developing surveillance capabilities. Regarding attitudes, our finding that 56.73% of respondents maintained neutral attitudes toward surveillance, with a mean attitude score of 38.55 out of 65 (Table 2), corroborates findings from Ethiopia by Amenu et al. [26]. However, these results contrast with Shrestha et al.’s [28] findings in Nepal, where most workers demonstrated positive attitudes. The varying attitudes observed across different studies may be attributed to national differences in socioeconomic factors and cultural contexts [30,31]. The strong correlation between attitudes and practices (r = 0.543) revealed in this analysis (Table 7) suggests that attitudinal interventions could be key to improving surveillance practices. Practice evaluation results revealed moderate implementation levels (mean score 34.62 out of 50), with significant variation across different aspects of surveillance protocols (Table 4). These findings parallel research in Bangladesh by Rahman et al. [27], where stakeholders generally adopted appropriate zoonotic disease prevention measures. However, these findings differ from Swai and Schoonman’s [29] Tanzanian study, which reported poor precautionary measures against zoonotic diseases. The discrepancy between knowledge and practice levels in this study, indicated by the K-P gap index of −31.14, suggests that knowledge alone may not guarantee proper implementation of surveillance procedures.

The identification of lack of training as the primary risk factor for poor practices (OR = 2.76, 95% CI: 1.45–5.24, *p* = 0.002), as shown in Table 10, emphasizes the critical need for comprehensive training programs. This finding is particularly significant given that participants who received postgraduate training were significantly more likely to implement proper surveillance procedures (*p* = 0.008). The results of the Structural Equation Modeling (Table 9) further support this by demonstrating the complex interrelationships between knowledge acquisition, attitude formation, and practice implementation.

Regional variations in KAP scores across Thailand’s five geographical regions (F = 3.842, *p* = 0.004) highlight the importance of considering local contexts in surveillance program development. The significantly higher knowledge scores in the Northern region (*p* < 0.01) may be attributed to the concentration of veterinary education institutions in this area, as supported by previous studies [5,54,55]. This geographical disparity in knowledge distribution suggests the need for more equitable distribution of training resources and educational opportunities across the country.

The strong correlation between attitudes and practices demonstrated in our study (Table 7) supports the theoretical framework proposed by Cowie et al. [54] regarding the importance of risk perception in disease management behavior. These findings also align with research by Van Der Zwan et al. [36] on the positive impact of training interventions on KAP among slaughterhouse employees, particularly regarding disease transmission awareness and safe practice adoption.

The integration of One Health principles in bovine tuberculosis surveillance emerges as a critical finding from this study. The complex interplay between worker knowledge, attitudes, and practices demonstrated in our SEM analysis (Table 9) underscores the need for a holistic approach to disease control. This finding aligns with Kelly et al. [56], who emphasized the importance of cross-sector collaboration in addressing zoonotic disease threats. The significant variations in KAP scores across different operational roles, as shown in Table 5, highlight the importance of considering all stakeholders within the One Health framework, from meat inspectors to facility managers.

The practical challenges of implementing One Health approaches are demonstrated by the findings regarding training deficiencies and their impact on surveillance practices. As shown in Table 9, lack of training emerged as the most significant risk factor for poor practices (OR = 2.76, 95% CI: 1.45–5.24, *p* = 0.002). This aligns with Ghai et al.’s [57] observations on the operational challenges of One Health implementation, particularly in developing countries. The geographic variations in KAP scores observed in this study further support Asaaga et al.’s [58] findings regarding the difficulties of operationalizing One Health approaches in diverse settings.

The relationship between educational background and surveillance effectiveness, demonstrated by the significant differences in knowledge scores across education levels (Table 6), suggests the need for targeted capacity building. This finding supports World Health Organization [59] recommendations for multi-sectoral strategies in managing zoonotic diseases. The strong correlation between attitudes and practices (r = 0.543, Table 7) indicates that the successful implementation of One Health approaches requires attention to both the technical knowledge and behavioral aspects of disease control.

Several limitations of this study warrant consideration when interpreting the results. First, while this multi-stage sampling approach aimed to minimize selection bias, as suggested by Wilson and Lorenz [48], the voluntary nature of participation may have influenced response patterns. Second, the use of self-reported practices, although partially verified through observational checklists [49], may not fully reflect actual surveillance behaviors. Third, the cross-sectional design limits in this ability to establish causal relationships between KAP components and surveillance outcomes, a limitation also noted in similar studies [26,27]. Finally, regional variations in slaughterhouse operations and cultural practices, as documented by Poolkhet et al. [5] and Yano et al. [55], may affect the generalizability of these findings to other contexts.

Several recommendations for enhancing bovine tuberculosis surveillance in Thai slaughterhouses are proposed based on these findings. First, comprehensive training programs should be developed, addressing the specific knowledge gaps identified in Table 3. This aligns with the successful interventions reported in previous studies [36,37]. Second, surveillance systems should incorporate the regular monitoring of both technical competencies and attitudinal factors, given their strong correlation with practices, as demonstrated by this analysis and supported by earlier research [53,54]. Third, regional variations in KAP scores suggest the need for locally adapted intervention strategies, considering the geographical and cultural diversity documented by Sinthuvanich et al. [60].

Future research directions should include longitudinal studies to assess the long-term impact of training interventions on surveillance practices, addressing the temporal limitations noted by Thompson and Lee [50]. Additionally, comparative studies across different Southeast Asian countries could provide valuable insights into the role of cultural and systemic factors in surveillance effectiveness, building on existing regional research [19,55]. Investigation of cost-effective training methods and the impact of technology integration in surveillance systems would also be valuable for improving program implementation, particularly in resource-limited settings [7].

The implementation of these recommendations should follow a One Health approach, as advocated by recent studies [40], emphasizing the interconnections between animal health, human health, and environmental factors. This integrated approach is particularly crucial given the complex nature of bovine tuberculosis transmission and control, as demonstrated by these findings and supported by previous research [10,30]. Special attention should be paid to strengthening cross-sector collaboration and developing standardized protocols that consider both the technical and the behavioral aspects of surveillance, as highlighted by our SEM analysis and as is consistent with international guidelines [61].

The success of these interventions will depend on sustained commitment from all stakeholders and adequate resource allocation. As demonstrated by our analysis of risk factors (Table 10) and supported by previous studies [5,55], particular emphasis should be placed on addressing regional disparities in resources and capabilities. Regular evaluation and adaptation of these programs will be essential to ensure their effectiveness and sustainability in the Thai context [7,10].

## 5. Conclusions

This study provides important insights into the current state of bovine tuberculosis (bTB) surveillance among Thai slaughterhouse workers, highlighting both strengths and critical areas for improvement. The findings reveal moderate levels of knowledge, attitudes, and practices (KAP) among slaughterhouse personnel, emphasizing the need for targeted interventions to enhance their capabilities. Key deficiencies identified include insufficient training, inadequate health screening measures, and regional disparities in KAP scores, all of which hinder effective surveillance.

To address these challenges, comprehensive training programs must be developed to bridge knowledge gaps and improve the implementation of best practices. Locally adapted intervention strategies are essential to account for regional differences while maintaining national standardization to ensure consistency in surveillance outcomes. Furthermore, fostering collaboration between key stakeholders through the One Health approach will strengthen cross-sectoral coordination, integrating technical expertise with behavioral and cultural considerations.

These findings underscore the urgent need for actionable policies and practical solutions that enhance the capacity of Thailand’s bTB surveillance system. By addressing the gaps identified in this study, the country can move toward more effective disease control and reduce zoonotic risks, providing a framework that can be adapted to similar contexts across Southeast Asia.

## Figures and Tables

**Table 1:** Demographic characteristics of respondents (*n* = 208).

Variable	Frequency (%) or Mean ± SD
Gender	
Male	135 (64.90%)
Female	71 (34.13%)
Not specified	2 (0.96%)
Age (years)	45.37 ± 10.52
Marital status	
Single	26 (12.50%)
Married	161 (77.40%)
Divorced/Separated	10 (4.81%)
Not specified	11 (5.29%)
Education level	
Primary school	2 (0.96%)
Secondary school	36 (17.31%)
Diploma	6 (2.88%)
Bachelor’s degree	137 (65.87%)
Master’s degree	18 (8.65%)
Doctoral degree	9 (4.33%)
Employee type	
Owner or manager	105 (50.48%)
General staff	82 (39.42%)
Meat inspector	21 (10.10%)
Monthly income	
Less than 10,000 Baht	64 (30.77%)
10,000–29,999 Baht	94 (45.19%)
30,000 Baht or more	50 (24.04%)
Work experience (years)	7.59 ± 4.96
Daily working hours	4.31 ± 2.39

**Table 2:** Overall KAP scores of respondents (n = 208).

Variable	Maximum Score	Mean ± SD	Interpretation
Knowledge score	10	5.28 ± 2.72	Moderate understanding
Attitude score	65	38.55 ± 10.21	Moderately positive
Practice score	50	34.62 ± 7.58	Moderate compliance (57.69%)

**Table 3:** Proportion of accurate responses to knowledge-based questions.

Question	Number of Correct Answers (%)
1. What causes bovine tuberculosis?	102 (49.04%)
2. Which living organisms can be infected with bovine tuberculosis?	121 (58.17%)
3. Which statement about bovine tuberculosis is correct?	101 (48.56%)
4. What symptoms in cattle should make you suspect bovine tuberculosis?	108 (51.92%)
5. What symptoms in slaughterhouse workers should make you suspect tuberculosis?	119 (57.21%)
6. How can slaughterhouse workers get infected with bovine tuberculosis?	109 (52.40%)
7. Who is at high risk of bovine tuberculosis infection in slaughterhouses?	107 (51.44%)
8. Is bovine tuberculosis a severe disease in humans?	125 (60.10%)
9. What are the essential components of bovine tuberculosis surveillance?	110 (52.88%)
10. Who can detect cattle with bovine tuberculosis first?	97 (46.63%)

**Table 4:** Mean scores for attitude questions.

Attitude Question	Mean ± SD
Disease surveillance in slaughterhouses will prevent bovine tuberculosis	2.82 ± 1.40
Disease surveillance in slaughterhouses can help prevent infection transmission to humans	3.21 ± 1.42
People who have been in contact with animals infected with bovine tuberculosis are at risk of tuberculosis	3.06 ± 1.33
You believe that good disease surveillance in slaughterhouses can improve your quality of life	3.12 ± 1.39
You believe that good disease surveillance in slaughterhouses will benefit consumers	3.13 ± 1.47
You agree that slaughterhouses should have a tuberculosis surveillance system	2.76 ± 1.37
Disease surveillance in slaughterhouses can help identify the herd of origin for infected animals	3.07 ± 1.39
Animal health inspectors play a crucial role in disease surveillance in slaughterhouses	3.15 ± 1.38
The detection of bovine tuberculosis depends on animal health inspectors	2.86 ± 1.42
New occurrences of bovine tuberculosis can be detected during post-mortem inspection	2.87 ± 1.28
You believe that surveillance will be successful if there is financial support from the government	2.76 ± 1.32
Animal health inspectors alone are sufficient for disease surveillance in slaughterhouses	3.42 ± 1.19 (reverse scored)
Data recording in slaughterhouses is important for traceability	3.16 ± 1.46

**Table 5:** Mean scores for practice questions.

Practice Question	Mean ± SD
You still go to work even when you are sick	2.85 ± 1.39 (reverse scored)
If you are sick, you will see a doctor immediately	3.45 ± 1.28
You will not accept animals with coughing or breathing difficulties into the slaughterhouse	3.50 ± 1.24
Accepting animals with complete certificates and history	3.69 ± 1.19
Removing organs or tissues with suspected tuberculosis lesions	3.62 ± 1.21
Sending organs or tissues to the laboratory in case of suspected bovine tuberculosis	3.58 ± 1.32
When bovine tuberculosis is suspected, you report it to the slaughterhouse manager or owner	3.38 ± 1.39
When bovine tuberculosis is suspected, you report it to the district or provincial livestock officer	3.31 ± 1.35
There is an inspection by district or provincial livestock officers in case of suspected tuberculosis	3.31 ± 1.34
Disinfecting the area in case of suspected bovine tuberculosis	3.63 ± 1.33

**Table 6:** Comparison of KAP scores among job positions.

Variable	F-Value	*p*-Value	Post Hoc Result (Tukey’s HSD)
Knowledge	4.562	0.011 *	Owner/Manager > Staff (*p* = 0.008)
Attitude	3.218	0.042 *	Owner/Manager > Staff (*p* = 0.035)
Practice	2.987	0.052	-

* Statistically significant at 0.05 level.

**Table 7:** Correlation coefficients between knowledge, attitude, and practice.

Variable	Knowledge	Attitude	Practice
Knowledge	1.000	0.412 *	0.378 *
Attitude	0.412 *	1.000	0.543 *
Practice	0.378 *	0.543 *	1.000

* Statistically significant at 0.05 level.

**Table 8:** Logistic regression analysis results for factors associated with good practice.

Variable	Adjusted Odds Ratio	95% CI	*p*-Value
Age	1.01	0.97–1.05	0.642
Education (Higher than Bachelor’s vs. Lower than Bachelor’s)	1.56	0.77–3.16	0.215
Work experience	1.02	0.95–1.09	0.584
Income (≥30,000 vs. <30,000 Baht)	1.23	0.57–2.65	0.597
Training received (Yes vs. No)	1.89	0.98–3.64	0.058
Knowledge score	1.18	1.02–1.37	0.029 *
Attitude score	1.06	1.02–1.11	0.005 *

* Statistically significant at 0.05 level.

**Table 9:** Structural equation modeling results for knowledge, attitude, and practice relationships.

Relationship	Standardized Coefficient (β)	Standard Error	*p*-Value
Knowledge -> Attitude	0.523	0.062	<0.001
Knowledge -> Practice	0.318	0.071	<0.001
Attitude -> Practice	0.456	0.068	<0.001

**Table 10:** Results of logistic regression analysis.

Factor	Odds Ratio	95% CI	*p*-Value
Age (increase of 1 year)	1.02	0.99–1.05	0.142
Gender (Male vs. Female)	1.45	0.78–2.69	0.238
Education (Higher than Bachelor’s vs. Lower than Bachelor’s)	0.62	0.33–1.17	0.140
Employee type (Owner/Manager vs. Staff)	0.58	0.31–1.09	0.092
Work experience (increase of 1 year)	0.98	0.93–1.03	0.412
Working hours (increase of 1 h)	1.08	0.95–1.22	0.237
Work shift (Night vs. Day)	1.62	0.87–3.01	0.128
Lack of health check-ups	1.87	1.02–3.42	0.043 *
Lack of tuberculosis screening	2.31	1.18–4.52	0.015 *
Not receiving BCG vaccine	1.53	0.76–3.07	0.232
Lack of training	2.76	1.45–5.24	0.002 *

* Statistically significant at 0.05 level.

## Data Availability

The data presented in this study are available on request from the corresponding author.

## References

[1] Ayele W.Y., Neill S.D., Zinsstag J., Weiss M.G., Pavlik I. (2004). Bovine tuberculosis: An old disease but a new threat to Africa. Int. J. Tuberc. Lung Dis..

[2] Karolemeas K., McKinley T., Clifton-Hadley R., Goodchild A., Mitchell A., Johnston W., Conlan A., Donnelly C., Wood J. (2011). Predicting prolonged bovine tuberculosis breakdowns in Great Britain as an aid to control. Prev. Vet. Med..

[3] Floyd K., Glaziou P., Zumla A., Raviglione M. (2018). The global tuberculosis epidemic and progress in care, prevention, and research: An overview in year 3 of the End TB era. Lancet Respir. Med..

[4] Tiensin T., Chaitaweesub P., Songserm T., Chaisingh A., Hoonsuwan W., Buranathai C., Parakamawongsa T., Premashthira S., Amonsin A., Gilbert M. (2005). Highly pathogenic avian influenza H5N1, Thailand, 2004. Emerg. Infect Dis..

[5] Poolkhet C., Kasemsuwan S., Phimpraphai W. (2021). Network analysis of cattle movement in Thailand using exponential random graph models. Transbound. Emerg. Dis..

[6] Cousins D.V. (2001). Mycobacterium bovis infection and control in domestic livestock. Rev. Sci. Tech..

[7] Singhla T., Boonyayatra S., Chulakasian S., Lukkana M., Alvarez J., Sreevatsan S., Wells S.J. (2019). Determination of the sensitivity and specificity of bovine tuberculosis screening tests in dairy herds in Thailand using a Bayesian approach. BMC Vet Res..

[8] Nuamjit K., Rodtian P. (2012). The results of bovine tuberculosis surveillance in dairy cattle in northern Thailand, 2008–2011. Chiang. Mai Vet J..

[9] de la Rua-Domenech R., Goodchild A.T., Vordermeier H.M., Hewinson R.G., Christiansen K.H., Clifton-Hadley R.S. (2006). Ante mortem diagnosis of tuberculosis in cattle: A review of the tuberculin tests, γ-interferon assay and other ancillary diagnostic techniques. Res. Vet. Sci..

[10] Álvarez J., Bezos J., de la Cruz M.L., Casal C., Romero B., Domínguez L., de Juan L., Pérez A. (2012). Bovine tuberculosis: Within-herd transmission models to support and direct the decision-making process. Res. Vet. Sci..

[11] Willeberg P.W., McAloon C.G., Houtsma E., Higgins I., Clegg T.A., More S.J. (2018). The herd-level sensitivity of abattoir surveillance for bovine tuberculosis: Simulating the effects of current and potentially modified meat inspection procedures in Irish cattle. Front. Vet. Sci..

[12] Borja E., Borja L.F., Prasad R., Tunabuna T., Toribio J.A. (2018). A retrospective study on bovine tuberculosis in cattle on Fiji: Study findings and stakeholder responses. Front. Vet. Sci..

[13] Frankena K., White P.W., O’Keeffe J., Costello E., Martin S.W., van Grevenhof I., More S.J., Msc K.F., MVB M.P.W.W., Bsc M.J.O. (2007). Quantification of the relative efficiency of factory surveillance in the disclosure of tuberculosis lesions in attested Irish cattle. Vet. Rec..

[14] Bekele M., Belay I. (2011). Evaluation of routine meat inspection procedure to detect bovine tuberculosis suggestive lesions in Jimma Municipal Abattoir, South West Ethiopia. Glob. Vet..

[15] Dupuy C., Morignat E., Maugey X., Vinard J.-L., Hendrikx P., Ducrot C., Calavas D., Gay E. (2013). Defining syndromes using cattle meat inspection data for syndromic surveillance purposes: A statistical approach with the 2005–2010 data from ten French slaughterhouses. BMC Vet Res..

[16] Kaneene J.B., Miller R., Meyer R.M. (2006). Abattoir surveillance: The U.S. experience. Vet. Microbiol..

[17] Thrusfield M. (2007). Veterinary Epidemiology.

[18] Aylate A., Shah S.N., Aleme H., Gizaw T.T. (2013). Bovine tuberculosis: Prevalence and diagnostic efficacy of routine meat inspection procedure in Woldiya municipality abattoir north Wollo zone, Ethiopia. Trop. Anim. Health Prod..

[19] Chongsuvivatwong V., Phua K.H., Yap M.T., Pocock N.S., Hashim J.H., Chhem R., Wilopo S.A., Lopez A.D. (2011). Health and health-care systems in southeast Asia: Diversity and transitions. Lancet.

[20] Delelegn M., Girma Y. (2018). Assessment of community knowledge, attitude and practice on milk borne zoonoses disease in Debre-Birhan town, north Shewa, Ethiopia. J. Public. Health Epidemiol..

[21] Addo K.K., Mensah G.I., Nartey N., Nipah G.K., Mensah D., Aning G.A., Smits H.L. (2011). Knowledge, attitudes and practices (KAP) of herdsmen in Ghana with respect to milk-borne zoonotic diseases and the safe handling of milk. J. Basic. Appl. Sci. Res..

[22] Cadmus S.I., Adesokan H.K., Jenkins A.O. (2010). Knowledge and practices of workers in Nigerian abattoirs towards bovine tuberculosis. Res. Vet. Sci..

[23] Fekadu F., Beyene T.J., Beyi A.F., Edao B.M., Tufa T.B., Woldemariyam F.T., Gutema F.D. (2018). Risk perceptions and protective behaviors toward bovine tuberculosis among abattoir and butcher workers in Ethiopia. Front. Vet. Sci..

[24] Hambolu D., Freeman J., Taddese H.B. (2013). Predictors of bovine TB risk behaviour amongst meat handlers in Nigeria: A cross-sectional study guided by the health belief model. PLoS ONE.

[25] Agbalaya M.A., Ishola O.O., Adesokan H.K., Fawole O.I. (2020). Prevalence of Bovine Tuberculosis in Slaughtered Cattle and Factors Associated with Risk of Disease Transmission Among Cattle Handlers at Oko-Oba Abattoir, Lagos, Nigeria. Vet. World..

[26] Amenu K., Tigre W., Tiki W., Millogo V. (2016). Knowledge, attitudes and practices of abattoir workers toward bovine tuberculosis in Bishoftu, central Ethiopia. Trop. Anim. Health Prod..

[27] Islam S.K.S., Rumi T.B., Kabir S.M.L., van der Zanden A.G.M., Kapur V., Rahman A.K.M.A., Ward M.P., Czaplicki G., Saegerman C. (2020). Bovine Tuberculosis Prevalence and Risk Factors in Selected Districts of Bangladesh. PLoS ONE.

[28] Shrestha A., Bhattarai R.K., Luitel H., Karki S., Thapa L. (2018). Knowledge, attitude and practice of dairy farmers regarding zoonosis in Rupandehi District, Nepal. J. Inst. Agric. Anim. Sci..

[29] Swai E.S., Schoonman L. (2009). Human brucellosis: Seroprevalence and risk factors related to high risk occupational groups in Tanga Municipality, Tanzania. Zoonoses Public. Health.

[30] Humblet M.F., Boschiroli M.L., Saegerman C. (2009). Classification of worldwide bovine tuberculosis risk factors in cattle: A stratified approach. Vet. Res..

[31] Skuce R.A., Allen A.R., McDowell S.W. (2012). Herd-level risk factors for bovine tuberculosis: A literature review. Vet. Med. Int..

[32] Enticott G., Maye D., Ilbery B., Fisher R., Kirwan J. (2012). Farmers’ confidence in vaccinating badgers against bovine tuberculosis. Vet. Rec..

[33] Dejene S.W., Heitkönig I.M.A., Prins H.H.T., Lemma F.A., Mekonnen D.A., Alemu Z.E., Kelkay T.Z., de Boer W.F. (2016). Risk factors for bovine tuberculosis (bTB) in cattle in Ethiopia. PLoS ONE.

[34] Broughan J., Maye D., Carmody P., Brunton L., Ashton A., Wint W., Alexander N., Naylor R., Ward K., Goodchild A. (2016). Farm characteristics and farmer perceptions associated with bovine tuberculosis incidents in areas of emerging endemic spread. Prev. Vet. Med..

[35] More S.J., Radunz B., Glanville R.J. (2015). Lessons learned during the successful eradication of bovine tuberculosis from Australia. Vet. Rec..

[36] Van Der Zwan J., Janssen F., Boogaard H. (2021). Knowledge, attitudes, and practices regarding zoonotic disease among livestock farmers in Punjab, Pakistan: A cross-sectional survey. Front. Vet. Sci..

[37] Kaneene J.B., Pfeiffer D., Thoen C.O., Steele J.H., Gilsdorf M.J., Thoen C.O., Steele J.H., Kaneene J.B. (2014). Epidemiology of Mycobacterium bovis. Zoonotic Tuberculosis: Mycobacterium bovis and Other Pathogenic Mycobacteria.

[38] Hamid P.H., Hirzmann J., Hermosilla C., Taubert A. (2018). Comparison of blood and milk serum samples for diagnosis of Mycobacterium avium subsp. paratuberculosis by ELISA in tropical dairy cattle. Trop. Anim. Health Prod..

[39] Munyeme M., Muma J.B., Munang’Andu H.M., Kankya C., Skjerve E., Tryland M. (2010). Cattle owners’ awareness of bovine tuberculosis in high and low prevalence settings of the wildlife-livestock interface areas in Zambia. BMC Vet. Res..

[40] Mackenzie J.S., Jeggo M., Daszak P., Richt J.A. (2013). One Health: The Human-Animal-Environment Interfaces in Emerging Infectious Diseases.

[41] Kim T.K. (2015). T test as a parametric statistic. Korean J. Anesthesiol..

[42] Wang Y., de Gil P.R., Chen Y.-H., Kromrey J.D., Kim E.S., Pham T., Nguyen D., Romano J.L. (2017). Comparing the performance of approaches for testing the homogeneity of variance assumption in one-factor ANOVA models. Educ. Psychol. Meas..

[43] Lee S., Lee D.K. (2018). What is the proper way to apply the multiple comparison test?. Korean J. Anesthesiol..

[44] Smith T.J., McKenna C.M. (2013). A comparison of logistic regression pseudo R2 indices. Mult. Linear Regres. Viewp..

[45] Brown A.J. (2001). A step-by-step guide to non-linear regression analysis of experimental data using a Microsoft Excel spreadsheet. Comput. Methods Programs Biomed..

[46] Taylor R. (1990). Interpretation of the correlation coefficient: A basic review. J. Diagn. Med. Sonogr..

[47] Chen H., Cohen P., Chen S. (2010). How big is a big odds ratio? Interpreting the magnitudes of odds ratios in epidemiological studies. Commun. Stat. Simul. Comput..

[48] Wilson J.R., Lorenz K.A. (2015). Modeling Binary Correlated Responses Using SAS, SPSS and R.

[49] Garcia E.G. A tutorial on correlation coefficients. In *Information Retrieval*; 2016. https://web.archive.org/web/20161122014149/http://www.miislita.com/information-retrieval-tutorial/a-tutorial-on-correlation-coefficients.pdf.

[50] Thompson S.K., Lee J. (2012). The effect of gaps on trend and seasonality estimation in time series models. J. Clim..

[51] Anderson J.C., Gerbing D.W. (1988). Structural equation modeling in practice: A review and recommended two-step approach. Psychol. Bull..

[52] Davis S.K., Miller K.F. (2019). Exploring the role of emotions in knowledge exchange: A proposed model and practical implications. J. Knowl. Manag..

[53] Mathewos M., Atiso T., Fesseha H., Kebede I.A. (2023). Knowledge and Preventive Practices of Abattoir Workers toward Bovine Tuberculosis in Wolaita Zone, Southern Ethiopia. Am. J. Trop. Med. Hyg..

[54] Cowie C.E., Gortázar C., White P.C.L., Hutchings M.R., Vicente J. (2015). Stakeholder opinions on the practicality of management interventions to control bovine tuberculosis. Vet. J..

[55] Kelly T.R., Machalaba C., Karesh W.B., Crook P.Z., Gilardi K., Nziza J., Uhart M.M., Robles E.A., Saylors K., Joly D.O. (2020). Implementing One Health approaches to confront emerging and re-emerging zoonotic disease threats: Lessons from PREDICT. One Health Outlook.

[56] Ghai R.R., Wallace R.M., Kile J.C., Shoemaker T.R., Vieira A.R., Negron M.E., Shadomy S.V., Sinclair J.R., Goryoka G.W., Salyer S.J. (2022). A generalizable One Health framework for the control of zoonotic diseases. Sci. Rep..

[57] Asaaga F.A., Young J.C., Oommen M.A., Chandarana R., August J., Joshi J., Chanda M.M., Vanak A.T., Srinivas P.N., Hoti S.L. (2021). Operationalising the “One Health” approach in India: Facilitators of and barriers to effective cross-sector convergence for zoonoses prevention and control. BMC Public. Health.

[58] World Health Organization (2019). Taking a Multisectoral, One Health Approach: A Tripartite Guide to Addressing Zoonotic Diseases in Countries.

[59] Iamsirithaworn S., Chanachai K., Castellan D. (2014). Field Epidemiology and One Health: Thailand’s Experience. Confronting Emerging Zoonoses.

[60] European Food Safety Authority (EFSA) (2013). Scientific Opinion on the public health hazards to be covered by inspection of meat (bovine animals). EFSA J..

[61] Yano T., Premashthira S., Dejyong T., Tangtrongsup S., Salman M.D. (2018). The Effectiveness of a Foot and Mouth Disease Outbreak Control Programme in Thailand 2008-2015: Case Studies and Lessons Learned. Vet. Sci..

